# Vulnerability analysis of the economic-social-ecological complex system of Island tourism destinations

**DOI:** 10.1371/journal.pone.0324714

**Published:** 2025-07-23

**Authors:** Mingya Zhang, Yetao Zhou, Senyao Sang

**Affiliations:** 1 School of Marxism, Zhejiang Pharmaceutical University, Ningbo, Zhejiang, China; 2 Department of Tourism Management, Zhengjiang Ocean University, Zhoushan, Zhejing, China; Fiji National University, FIJI

## Abstract

Exploring the vulnerability characteristics of the economic-social-ecological complex system of island tourism destinations can provide better theoretical and practical guidance for the sustainable development of island tourism destinations. Taking Zhoushan, which is a famous island tourism destination in China, as a case, based on the vulnerability analysis framework of exposure, sensitivity, and adaptive capacity, this paper employs the entropy value method, comprehensive index method, system coordination degree model, and obstacle model to quantitatively measure the vulnerability of the economic-social-ecological complex system of Zhoushan from 2011 to 2022. It analyzes the spatial and temporal heterogeneity characteristics of the complex system`s vulnerability and the obstacle factors in the four administrative regions that make up the case site. The research results reveal that the vulnerability of Zhoushan’s economic-social-ecological complex system shows a decreasing trend, indicating its ability to withstand external disturbances continuously increases. However, the decline in the coordinated development of vulnerability reveals the imbalance in the development of internal systems among the four different regions. Furthermore, the study finds that the degree of obstacles to the ecological system is relatively high at the system level. In contrast, at the indicator level, the main obstacle factors are the total import and export trade, density of the tourism economy, construction land ratio, island land and sea area coefficient, and tourist density. Based on these findings, the study suggests deepening trans-regional cooperation, paying attention to constructing ecological systems and promoting the coordinated development of the island tourism destination complex system to reduce the overall vulnerability of island tourism destinations.

## 1. Introduction

With the rapid development of economic globalization and the growing scarcity of terrestrial natural resources, the development of the ocean economy has become a focus for many countries’ economic growth, increasingly highlighting the strategic importance of the maritime domain [1]. Islands are strategic strongholds for exploiting marine resources and are crucial to the “human-ocean coupling system.” Given the vulnerability of the social cultures and natural ecosystems, islands are confronted with a dichotomy between economic development and social and ecological sustainability [2]. Because of this, as a valid means for achieving coordinated economic, social, and ecological development, island tourism has gained favor among many island planners by fostering differentiated islandness-based island branding [3], such as Seychelles, the Caribbean, and Hawaii [4].

As imagined utopian places, islands are highly preferred by tourists seeking authenticity and others. Taking Zhoushan Islands as an example, which is China’s most famous island tourism destination, it received a cumulative 12.14 million visitors in the first three quarters of 2023. However, while tourism brings development opportunities and economic benefits to island regions, the high density of population flow and over-tourism development have caused such negative impacts on the original social, economic, and ecological system [5–7], exacerbating the “man-land” conflict. Thus, how to construct a dynamically coordinated economic-social-ecological complex system under the context of tourism development and then construct the harmonious “man-land” relationship has become an important issue.

The economic-social-ecological complex system of island tourism destinations is a man-land relationship system with tourism activities as the main interference. It has the characteristics of complexity, dynamics, and multi-scale. It is essential to analyze the complex spatial and temporal evolution process from the perspectives of vulnerability, adaptive capacity, and resilience and to explore the factors influencing conflicts among subsystems [8].

Vulnerability is the possibility of a system being exposed to adverse impacts or suffering damage [9,10]. Originating from natural disaster studies [11], it is related to social groups’ exposure, sensitivity, and coping capacity when facing disasters [12,13]. It is the attribute of a system prone to structural and functional changes due to its sensitive reaction and lack of coping capacity when subjected to internal and external disturbances. Vulnerability has now been widely applied in various fields such as ecology [14,15], climate change [16,17], and sustainability science [18], which has evolved from a single-dimensional concept of natural vulnerability to a multi-dimensional and comprehensive concept [19], covering elements such as sensitivity, coping capacity, and exposure, and involving economic, social, and ecological dimensions [20]. With the continuous improvement of the theoretical system, the research methods of vulnerability have shifted from qualitative evaluation methods to quantitative methods such as set pair analysis method, comprehensive index methods, and 3S technologies [21–23]. Additionally, scholars adopt different analytical frameworks, such as “sensitivity-coping capacity” [24], “exposure-sensitivity-adaptive capacity” [25, 26], and “pressure-state-response” [27], to conduct empirical analyses on research areas at different scales.

The previous studies pointed out that one of the seven core issues in sustainability research is the study of vulnerability or resilience in specific regions [28]. Terrestrial systems such as forest ecosystems [29], grassland ecosystems [30], and urban systems [31] have been the focus of scholars’ research over the past two decades, and comprehensive evaluation systems have been formed. However, research on the vulnerability of island regions needs to be further revealed. Island regions are susceptible to natural disturbances such as climate change and are sensitive to the development of the marine economy [32]. Therefore, conducting vulnerability research on the economic-social-ecological complex system of island tourism destinations has significant practical value.

This study establishes a vulnerability index evaluation system for island tourism destinations based on the vulnerability analysis framework by taking the Zhoushan Archipelago in China as a case. It quantitatively measures the vulnerability of the economic-social-ecological complex system from 2011 to 2022 in four administrative regions in the Zhoushan Archipelago, including Dinghai, Putuo, Daishan, and Shengsi. Also, the research explores the spatial and temporal heterogeneity of the vulnerability of the three subsystems in the development process of Zhoushan as an island tourism destination. Furthermore, the study employs the obstacle model to diagnose the factors causing spatial and temporal heterogeneity in vulnerability. The research explores the formulation of symbiotic development strategies for the economic-social-ecological complex system of island tourism destinations.

## 2. Materials and methods

### 2.1. Study area

The Zhoushan Archipelago is located off the southeast coast of China and is administratively located in the city of Zhoushan. It borders the East China Sea to the east, Hangzhou Bay to the west, and Shanghai to the north. Composed of over 2,085 scattered islands, the archipelago covers 22,200 square kilometers, with a maritime area of 20,800 square kilometers. Zhoushan governs four administrative districts: Dinghai, Putuo, Daishan, and Shengsi. By 2022, the permanent population of Zhoushan was 1.17 million, with a population density of 802 people per square kilometer, resulting in a strained relationship between man and land and significant pressure on resource and environmental carrying capacity.

The Zhoushan Archipelago boasts tourism resources that combine islandscape, marine culture, and Buddhist culture, including world-renowned island tourist destinations such as Putuo Mountain and the Shengsi Islands. Relying on its unique tourism resources, the tourism industry in Zhoushan has experienced rapid development. However, the conduct of tourism activities has disrupted the economic-social-ecological complex system, which is not limited to the inherent spatial transformation and environmental degradation caused by tourism development but also includes the gentrification of islands due to over-tourism and sociocultural changes. The external environmental impacts, internal instability factors, and the inherent limitations of island space have made Zhoushan exhibit pronounced vulnerability characteristics.

### 2.2. Evaluation index

Polsky pointed out that vulnerability consisted of three main aspects: exposure to specific social or environmental stresses, sensitivity, and adaptive capacity [33]. If a system has low exposure, low sensitivity, or high adaptive capacity, it can persist under threat or pressure over the long term. Exposure refers to the degree to which a study unit is subjected to particular environmental or social stresses, indicating how vulnerable the human-environment system may be to hazardous factors and reflecting the extent of external disturbance or pressure [34]. Sensitivity denotes the extent to which an exposed unit is susceptible to positive or negative impacts from stress, pressure, or threats, illustrating the degree to which the stressor affects the system’s current state and describing a set of conditions or attributes that regulate the system’s short-term inclination to be affected after exposure [35]. Adaptive capacity refers to the self-regulating ability of the system after disturbance or pressure, as well as the capacity of human actions to restore the system to its original structural level or state [36]. Exposure and sensitivity jointly define the potential impact of stress sources, while adaptive capacity determines the ultimate effect. Thus, vulnerability is jointly determined by stressors’ potential and ultimate impacts. Drawing upon prior research [37,38], this paper decomposes vulnerability into exposure, sensitivity, and adaptive capacity, selecting 33 indicators across the economic, social, and ecological dimensions to establish a vulnerability assessment indicator system for the economic-social-ecological complex system of island tourism destinations (Table 1). The data are mainly derived from the panel data of 12 years (2011–2022).

**Table 1:** Vulnerability assessment indicator system for the economic-social-ecological complex system of island tourism destinations.

System layer	Criterion	Index layer	Index properties	Indicator description
**Economic system vulnerability**	Exposure	A1 Proportion of tourism revenue in GDP	+	Measure the status of the tourism industry in the national economy.
		A2 Elasticity coefficient of tourism growth	+	Measure the elasticity pressure of tourism growth on economic growth.
		A3 Density of tourism economy	+	Measure the development of the tourism economy
	Sensitivity	A4 Urban-rural income ratio	+	Measure the urban-rural income gap
		A5 Service output value ratio	–	Measure the structural development of the tertiary industry
		A6 Economic growth rate	+	Measure economic development efficiency
		A7 Financial self-sufficiency rate	–	Measure the fiscal self-sufficiency of the island
	Adaptive capacity	A8 Per capita annual fiscal revenue	+	Measure the economic strength of the island government
		A9 Disposable income	+	Measure the disposable income level of island residents
		A10 Total import and export trade of the island	+	Measure the degree of openness of the island
		A11 Growth rate of investment in fixed assets	+	Measure the material foundation and operational capacity.
**Social system** **vulnerability**	Exposure	B1 Tourist density	+	Measure the tourist pressure on the island
		B2 Island urbanization rate	+	Measure the urbanization process of the island
		B3 Ratio of tourists to residents	+	Measure the population structure of the island
	Sensitivity	B4 Engel co-efficient	+	Measure the ability to optimize the consumption structure.
		B5 Registered urban unemployment rate.	+	Measure the employment stability of island residents
		B6 Natural growth rate of population	+	Measure the growth rate of population pressure on the island
		B7 Population density	+	Measure the population pressure on the island
	Adaptive capacity	B8 Fiscal expenditure on culture, sports, tourism, and media	+	Measure the support for the island’s tourism industry
		B9 Number of travel agencies on the island	+	Measure the reception capacity of the island’s tourism
		B10 Per capita post and telecommunications business	+	Measure the island’s external communication capability
		B11 Island road network density	+	Measure the island’s road accessibility index.
**Ecological** **system** **vulnerability**	Exposure	C1 Solid waste emissions per unit of output value	+	Measure the pressure of solid waste discharge on the island
		C2 Wastewater discharge per unit output value	+	Measure the pressure of wastewater discharge on the island.
		C3 Electricity consumption per unit GDP	+	Measure the system’s electricity consumption
		C4 Proportion of construction land	+	Measure the pressure of land use
	Sensitivity	C5 Proportion of good air quality days	–	Measure the air quality of the island
		C6 Water quality in coastal waters	–	Measure the ecological environment of the sea area
		C7 Island land and sea area coefficient	+	Measure the resource endowment of the island
	Adaptive capacity	C8 Per capita available water resources	+	Measure the per capita water resources situation.
		C9 Per capita coastline length	+	Measure the status of the coastline
		C10 Energy consumption reduction rate per unit of GDP	+	Measure the system’s energy consumption pressure
		C11 The proportion of fiscal expenditure on environmental protection	+	Measure the intensity of ecological environment protection.

### 2.3. Methods

#### 2.3.1. Entropy weight method.

The vulnerability of island tourism destinations’ economic, social, and ecological systems varies due to different impacts from individual indicators. Hence, distinct weights should be assigned to each indicator. Considering these indicators’ diverse positive and negative influences on vulnerability, this study applies standardization through the extreme difference method to normalize the raw data. The entropy weight method, an objective weighting approach, assigns weights based on the variability or entropy of the indicators [39]. The following specific steps were taken in this research to calculate dimension indices and vulnerability indices using a weighted composite index method:

Indicator Selection: Let there be h years, m regions, and n indicators, where$X_{\lambda ij}$ represents the value of the $j^{th}$ indicator for the $i^{th}$ region in the $\lambda^{th}$ year.

Data standardization: In order to eliminate the influence of different dimensions on the overall evaluation, the range standardization method is used to deal with the original data, and the positive and negative indexes are calculated respectively.

For positive indicators:


$${X^{\prime }}_{\lambda ij}=\frac{x_{\lambda ij}-x_{\min}}{x_{\max}-x_{\min}}$$ (1)

For negative indicators:


$${X^{\prime }}_{\lambda ij}=\frac{x_{\max}-x_{\lambda ij}}{x_{\max}-x_{\min}}$$ (2)

Where ${X^{\prime }}_{\lambda ij}$ represents standardized values for different indicators, $x_{\max}$, $x_{\min}$ represents the maximum or minimum of the raw value of the first indicator, respectively.

Indicator Normalization: Standardize the units of measurement for all indicators and calculate the proportion of the value of the $j^{th}$ indicator for the $i^{th}$ region in the $\lambda^{th}$ year.


$$P_{\lambda ij}={x^{\prime }}_{\lambda ij}/\sum_{\lambda =1}^{h}\sum_{i=1}^{m}{x^{\prime }}_{\lambda ij}$$ (3)

Calculate the entropy value of each indicator.


$$e_{j}=-k\sum_{\lambda =1}^{h}\sum_{i=1}^{m}\left(P_{\lambda ij}\times \ln P_{\lambda ij}\right)$$ (4)

Calculate the entropy redundancy of each indicator.


$$d_{j}=1-e_{j}$$ (5)

Calculate the weights of the indicators.


$$w_{j}=d_{j}/\sum_{j=1}^{n}d_{j}$$ (6)

Calculate the index for each dimension: The comprehensive index method is based on each dimension’s specific constituent indicator factors, so it can effectively reflect the relative vulnerability of each component unit and serve as a decisive reference for further prediction. This paper uses the comprehensive index method to calculate the index values of each dimension of vulnerability.


$$\begin{array}{c} S_{\lambda i}=\sum_{j=1}^{n}\left(w_{j}{x^{\prime }}_{\lambda ij}\right) \end{array}$$ (7)

Where$S_{\lambda i}$ represents Exposure Index(E), sensitivity index(S), Adaptive capacity index(AC).

#### 2.3.2 Vulnerability calculation method.

Vulnerability can be expressed as a function of exposure, sensitivity, and adaptive capacity. Following the research by Chen Jia et al. [40], this study selects indicators representing the three dimensions of exposure, sensitivity, and adaptive capacity to construct an evaluation model for the vulnerability of the economic-social-ecological complex systems of island tourism destinations. The model is as follows:


V=E+S−AC (8)

In the formula, V represents the vulnerability index, E represents the exposure index, S represents the sensitivity index, and AC represents the adaptive capacity index. The larger the value of V, the greater the system’s vulnerability.

#### 2.3.3 Coordination degree model.....

The coordination degree model is introduced to further study the coordinated relationship among the social, economic, and ecological systems and better reflect the state of regional vulnerability [41, 42]. The binary system coordination degree model is as follows:


$$C={\left\{\frac{u_{1}u_{2}}{{\left[(u_{1}+u_{2})/2\right]}^{2}}\right\}}^{\frac{1}{2}}$$

$$T=\alpha u_{1}+\beta u_{2}$$

$$D=\sqrt{C\times T}$$ (9)

Based on the coupling mechanism of the two systems, a ternary system coordination development degree model is constructed:


$$C^{\prime }={\left\{\frac{u_{1}u_{2}u_{3}}{{\left[\left(u_{1}+u_{2}+u_{3}\right)/3\right]}^{3}}\right\}}^{\frac{1}{3}}$$

$$T^{\prime }=\alpha u_{1}+\beta u_{2}+\gamma u_{3}$$

$$D^{\prime }=\sqrt{C^{\prime }\times T^{\prime }}$$ (10)

Where C is the coupling degree, D is the coupling coordination degree, and T is the composite ecological system coordination development index.

#### 2.3.4. Obstacle factor diagnosis method.

To further analyze the indicators that significantly impact the vulnerability of the economic-social-ecological complex system of island tourist destinations from 2011 to 2022, the obstacle factor analysis is introduced to calculate the influence of each indicator on the complex system vulnerability of island tourist destinations. By ranking the obstacle degrees, the primary and secondary relationships of each obstacle factor and their impact on system vulnerability are determined [43]. The formula for calculating the obstacle degree is as follows:


$$Y_{\lambda ij}=1-X_{\lambda ij}^{\prime }$$

$$A_{j}=\frac{w_{j}Y_{\lambda ij}}{\sum_{j=1}^{n}w_{j}Y_{\lambda ij}}\times 100\;\%$$ (11)

In the formula: The obstacle degree $A_{j}$ represents the degree of influence of the $j^{th}$ indicator on the system vulnerability. The higher the obstacle degree of an indicator, the greater its influence.

## 3. Results

### 3.1. Vulnerability of each subsystem

**Fig 1:**
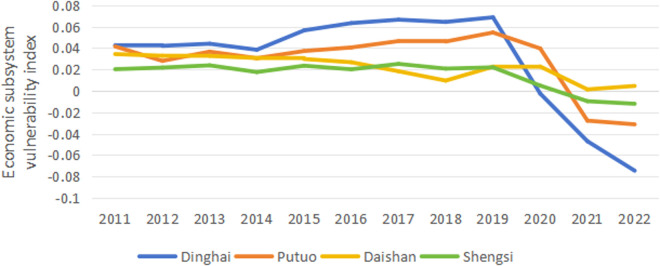
Trends of economic system vulnerability changes.

**Fig 2:**
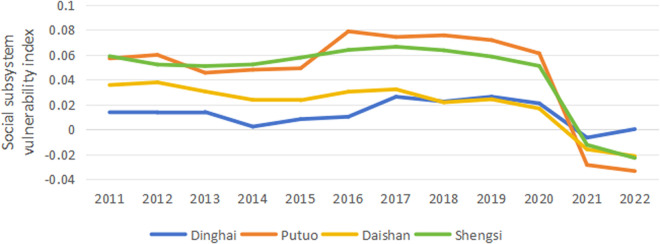
Trends of social system vulnerability changes.

**Fig 3:**
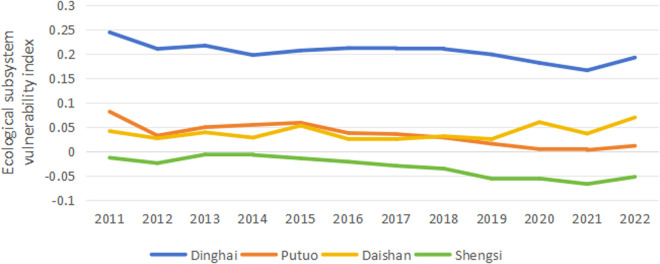
Trends of ecosystem vulnerability changes.

The vulnerability index calculation formula calculates the vulnerability indexes of the economic, social, and ecological subsystems in each region of Zhoushan from 2011 to 2022. By drawing line charts (Figs 1-3), further analysis is conducted on the temporal characteristics of the vulnerability indexes. As a result, the vulnerability of the economic and social subsystems exhibits a clear downward trend. In contrast, the change trend in the ecological subsystem’s vulnerability is relatively stable.

#### 3.3.1. Economic subsystem.

The trend of economic system vulnerability is shown in Fig 1. The trends of the four regions are roughly similar, reaching a peak around 2019 and then showing a general downward trend. This indicates that the overall economic system’s ability to withstand risks in Zhoushan has improved in recent years. In terms of the degree of vulnerability, from 2011 to 2019, the vulnerability of Dinghai is generally higher than that of the other three regions, followed by Putuo, with Shengsi and Daishan being relatively lower. This suggests that the economic systems of Dinghai and Putuo are more susceptible to external pressures. Furthermore, Dinghai has the most significant fluctuation in vulnerability values, sharply decreasing after peaking in 2019 and falling to the lowest in 2022. Putuo experiences a similar, albeit slightly less extreme, rise and fall. In contrast, Daishan and Shengsi have relatively smooth fluctuations. In terms of improvement, since 2020, Dinghai and Putuo have shown the most significant enhancements in vulnerability, with their values turning from positive to negative and experiencing a substantial decline. Shengsi also reveals a change from positive to negative, but the decrease is relatively minor. Overall, there are specific differences in the economic system vulnerability among the regions of Zhoushan, with Dinghai and Putuo having relatively higher economic vulnerability between 2011 and 2019, and the vulnerability of Daishan and Shengsi has been at a generally lower level.

#### 3.1.2. Social subsystem.

As shown in Fig 2, the overall trend of social vulnerability in various regions of Zhoushan is similar. It peaks during 2017−2019 and then begins to decline, especially after 2021, when the rate of decline significantly increases, turning from positive to negative values. This indicates that in recent years, the overall capacity of Zhoushan’s social system to resist risks has been enhanced. Unlike the economic system, from 2011 to 2020, the social system vulnerability of Putuo is generally higher than the other three regions. The vulnerability of Putuo’s social system shows a fluctuating upward trend from 2011 to 2018, reaching a peak of 0.079 in 2016 and then continuously declining after 2019, falling to a low of −0.033 in 2022, reflecting significant improvement in the social system’s vulnerability of Putuo in recent years. The following highest social system vulnerability is in Shengsi, which generally shows an upward trend from 2011 to 2017, reaching a peak of 0.066 in 2017 and declining year by year, falling to −0.023 in 2022. This indicates that the social system’s capacity to resist risks in Shengsi has continuously improved in recent years. The social system vulnerability indices of Dinghai and Daishan are relatively low and are in a general state of decline from 2011 to 2022. Overall, the social system vulnerability indices of various regions in Zhoushan are in a state of decline, and the overall capacity of the social system to resist risks has been enhanced.

#### 3.1.3 Ecological subsystem.

From the trend of ecosystem vulnerability (Fig 3), it can be seen that from 2011 to 2022, the ecological subsystem vulnerability index of Dinghai is significantly higher than that of Putuo, Daishan, and Shengsi. This indicates that, compared to the other three regions, the ecosystem in Dinghai is more vulnerable. From 2011 to 2022, Putuo’s ecological system vulnerability index generally shows a downward trend, decreasing from 0.081 in 2011 to 0.011 in 2022. Meanwhile, the ecological system vulnerability of Daishan shows a fluctuating upward trend during the period from 2011 to 2022, rising from 0.041 in 2011 to 0.069 in 2022, an increase of 69.1%, which indicates that the ecological system of Daishan faces increased risks and pressures. Moreover, the ecological system vulnerability index of Shengsi is consistently negative from 2011 to 2022. It shows a fluctuating downward trend, decreasing from −0.014 in 2011 to −0.053 in 2022, which demonstrates that the ecological system of Shengsi has a stronger resistance to disturbances, higher environmental quality, and faces relatively lower ecological risks.

### 3.2. Characteristics of complex system vulnerability

#### 3.2.1. Temporal heterogeneity.

**Fig 4:**
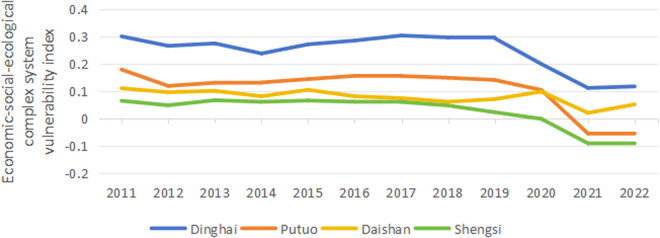
Trends of vulnerability changes in economic-social-ecological complex system.

The vulnerability of the complex system is the result of the joint action of regional economic, social, and ecological systems. Fig 4 shows the trend and regional differences in the vulnerability index of the economic-social-ecological complex system in Zhoushan from 2011 to 2022. The vulnerability index of each region shows a downward trend as a whole, indicating that the vulnerability of the economic-social-ecological complex system in each region continues to decrease, the development stability of the economic-social-ecological complex system gradually improves, and the probability of loss under the interference of tourism activities and human activities gradually decreases. The vulnerability index of Dinghai is the highest, but its vulnerability index generally shows a downward trend, from 0.3 in 2011 to 0.118 in 2022, a decrease of 60.8%. There is a slight rebound in 2013 and 2017. Still, the overall trend is better, indicating that the anti-interference ability of the economic-social-ecological complex system in Dinghai is gradually enhanced. The vulnerability index of Putuo shows an evolution trend of rising first and then falling. It shows an upward trend from 2011 to 2016, peaking at 0.156 in 2016, and then decreases year by year. It turns negative in 2021 and fell to −0.054 in 2022, indicating that the vulnerability of the economic and social ecosystem in Putuo has been rapidly improved in recent years. The vulnerability index of Daishan decreases from 0.111 in 2011 to 0.052 in 2022, and the anti-interference ability is generally enhanced. However, there has been a slight rebound since 2018. The vulnerability index of Shengsi shows a continuous downward trend, from 0.065 in 2011 to −0.088 in 2022, from positive to negative, and there is no rebound during the period, indicating that the vulnerability of its complex system was steadily reduced.

#### 3.2.2. Spatial heterogeneity.

Based on the results of the vulnerability index of island tourism destinations in Zhoushan from 2011 to 2022, this paper uses the natural breaks method of ArcGIS [44,45] to classify the vulnerability of each region. It obtains three levels of vulnerability: low, medium, and high. First of all, the vulnerability index is evaluated based on the vulnerability level standard of 2011, as shown in Table 2.

**Table 2:** Vulnerability index classification criteria.

Vulnerability Level	First Level	Second Level	Third Level
	Low Vulnerability	Medium Vulnerability	High Vulnerability
Vulnerability Index	< 0.110812	0.110813-0.179244	> 0.179245

**Fig 5:**
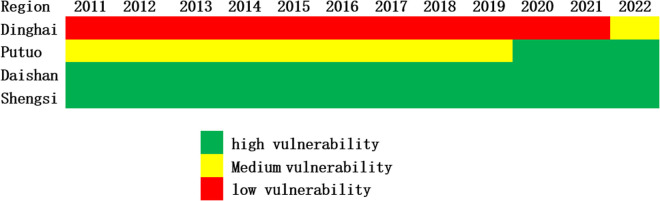
The spatial and temporal heterogeneity of vulnerability in Zhoushan.

As shown in Fig 5, image analysis from 2011 to 2022 indicates that the overall low vulnerability area is gradually replacing the medium vulnerability area, while the medium vulnerability area is replacing the high vulnerability area. This trend shows that the number of high-vulnerability regions is decreasing, and the number of low-vulnerability areas is increasing. This change indicates that Zhoushan’s economic-social-ecological complex system is developing more positively.

Although the economic-social-ecological complex system of Dinghai has been in a relatively high vulnerability state for a long time, the vulnerability index of its economic and ecological subsystems is relatively high, which makes the overall vulnerability index of the region higher. However, it is worth noting that the Dinghai has gradually evolved from a highly vulnerable state to a moderately vulnerable state. Similarly, Putuo has progressively evolved from a fragile to a low-fragile state. In addition, the economic-social-ecological complex system of Daishan and Shengsi remains in a low-vulnerability state. It is relatively stable, which indicates that the vulnerability of these two regions fluctuated less, reflecting the coordinated development of the man-land relations.

### 3.3. Analysis of the coordination degree

**Fig 6:**
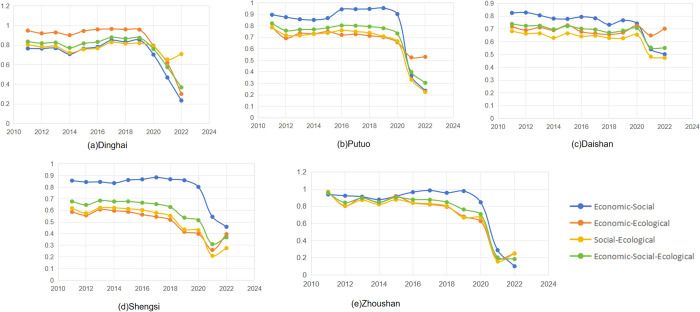
Trends of vulnerability coordination in systems.

Regarding the classification of coordination types, this paper categorizes the system’s coordination development status into ten major categories, as shown in Table 3. Based on the coordination model, this paper calculates and analyzes the temporal heterogeneity of the binary and ternary coordinated degree of the internal economy, society, and ecological subsystems of the four regions and their overall vulnerability in Zhoushan from 2011 to 2022 (Fig 6), and divides the coordination degree types. The results are shown in Table 4.

**Table 3:** Classification level of coordination degree.

Coordinated development degree	Type	Level	Coordinated development degree	Type	Level
[0.0,0.1)	Extreme Disorder	Ⅰ	[0.5,0.6)	Barely Coordination	Ⅵ
[0.1,0.2)	Severe Disorder	Ⅱ	[0.6,0.7)	Primary Coordination	Ⅶ
[0.2,0.3)	Moderate Disorder	Ⅲ	[0.7,0.8)	Intermediate Coordination	Ⅷ
[0.3,0.4)	Mild Disorder	Ⅳ	[0.8,0.9)	Good Coordination	Ⅸ
[0.4,0.5)	On the Verge of Disorder	Ⅴ	[0.9,1.0]	High-Quality Coordination	Ⅻ

**Table 4:** Types of Internal Coordinated Development of Vulnerability Systems in Zhoushan.

Region	System	2011	2012	2013	2014	2015	2016	2017	2018	2019	2020	2021	2022
**Dinghai**	Economic-Social	Ⅷ	Ⅷ	Ⅷ	Ⅷ	Ⅷ	Ⅷ	Ⅸ	Ⅸ	Ⅸ	Ⅷ	Ⅴ	Ⅲ
	Economic-Ecological	Ⅻ	Ⅻ	Ⅻ	Ⅻ	Ⅻ	Ⅻ	Ⅻ	Ⅻ	Ⅻ	Ⅷ	Ⅶ	Ⅳ
	Social-Ecological	Ⅸ	Ⅷ	Ⅷ	Ⅷ	Ⅷ	Ⅷ	Ⅸ	Ⅸ	Ⅸ	Ⅷ	Ⅶ	Ⅷ
	Economic-Social-Ecological	Ⅸ	Ⅸ	Ⅸ	Ⅷ	Ⅸ	Ⅸ	Ⅸ	Ⅸ	Ⅸ	Ⅷ	Ⅵ	Ⅳ
**Putuo**	Economic-Social	Ⅸ	Ⅸ	Ⅸ	Ⅸ	Ⅸ	Ⅻ	Ⅻ	Ⅻ	Ⅻ	Ⅻ	Ⅳ	Ⅲ
	Economic-Ecological	Ⅷ	Ⅶ	Ⅷ	Ⅷ	Ⅷ	Ⅷ	Ⅷ	Ⅷ	Ⅷ	Ⅶ	Ⅵ	Ⅵ
	Social-Ecological	Ⅷ	Ⅷ	Ⅷ	Ⅷ	Ⅷ	Ⅷ	Ⅷ	Ⅷ	Ⅷ	Ⅶ	Ⅳ	Ⅲ
	Economic-Social-Ecological	Ⅸ	Ⅷ	Ⅷ	Ⅷ	Ⅷ	Ⅸ	Ⅸ	Ⅷ	Ⅷ	Ⅷ	Ⅳ	Ⅳ
**Daishan**	Economic-Social	Ⅸ	Ⅸ	Ⅸ	Ⅷ	Ⅷ	Ⅷ	Ⅷ	Ⅷ	Ⅷ	Ⅷ	Ⅵ	Ⅵ
	Economic-Ecological	Ⅷ	Ⅶ	Ⅷ	Ⅶ	Ⅷ	Ⅶ	Ⅶ	Ⅶ	Ⅶ	Ⅷ	Ⅶ	Ⅷ
	Social-Ecological	Ⅶ	Ⅶ	Ⅶ	Ⅶ	Ⅶ	Ⅶ	Ⅶ	Ⅶ	Ⅶ	Ⅶ	Ⅴ	Ⅴ
	Economic-Social-Ecological	Ⅷ	Ⅷ	Ⅷ	Ⅶ	Ⅷ	Ⅷ	Ⅶ	Ⅶ	Ⅶ	Ⅷ	Ⅵ	Ⅵ
**Shengsi**	Economic-Social	Ⅵ	Ⅵ	Ⅶ	Ⅵ	Ⅵ	Ⅵ	Ⅵ	Ⅵ	Ⅴ	Ⅳ	Ⅲ	Ⅳ
	Economic-Ecological	Ⅵ	Ⅵ	Ⅶ	Ⅵ	Ⅵ	Ⅵ	Ⅵ	Ⅵ	Ⅴ	Ⅳ	Ⅲ	Ⅳ
	Social-Ecological	Ⅶ	Ⅵ	Ⅶ	Ⅶ	Ⅶ	Ⅶ	Ⅵ	Ⅵ	Ⅴ	Ⅴ	Ⅲ	Ⅲ
	Economic-Social-Ecological	Ⅶ	Ⅶ	Ⅶ	Ⅶ	Ⅶ	Ⅶ	Ⅶ	Ⅶ	Ⅵ	Ⅵ	Ⅳ	Ⅳ
**Zhoushan**	Economic-Social	Ⅻ	Ⅻ	Ⅻ	Ⅸ	Ⅻ	Ⅻ	Ⅻ	Ⅻ	Ⅻ	Ⅸ	Ⅲ	Ⅱ
	Economic-Ecological	Ⅻ	Ⅸ	Ⅸ	Ⅸ	Ⅻ	Ⅸ	Ⅸ	Ⅷ	Ⅶ	Ⅶ	Ⅱ	Ⅲ
	Social-Ecological	Ⅻ	Ⅸ	Ⅸ	Ⅸ	Ⅸ	Ⅸ	Ⅸ	Ⅸ	Ⅶ	Ⅶ	Ⅱ	Ⅲ
	Economic-Social-Ecological	Ⅻ	Ⅸ	Ⅸ	Ⅸ	Ⅻ	Ⅸ	Ⅸ	Ⅸ	Ⅷ	Ⅷ	Ⅲ	Ⅱ

From the binary structure analysis, it is concluded that the vulnerability coordination degree of the economic-social binary interaction system in Zhoushan from 2011 to 2022 experiences a transition from high-quality coordination to good coordination to moderate disorder to severe disorder, with the synergistic effect of social and economic development gradually decreasing; the vulnerability coordination degree of the economic-ecological binary interaction system from 2011 to 2022 undergoes a transition from high-quality coordination to good coordination to intermediate coordination to primary coordination to severe disorder to moderate disorder, with the synergistic effect of economic development and ecological protection decreasing; the vulnerability coordination degree of the social-ecological binary interaction system from 2011 to 2022 experiences a transition from high-quality coordination to good coordination to primary coordination to severe disorder to moderate disorder, with the synergistic effect of social development and ecological protection being poor. Regionally, the internal economic-social, economic-ecological, and social-ecological binary interaction systems in the four regions of Zhoushan mostly evolve from an earlier stage of coordination to a level of disorder. In phases from 2011 to 2018, the highest level of vulnerability coordination in Zhoushan, as well as in Putuo, Daishan, and Shengsi, is in the economic-social system, with the most substantial synergistic effect of social and economic development during this period, while in Dinghai during this period, the highest level of vulnerability coordination is in the economic-ecological system, marking a phase when the vulnerability coordination levels of all three binary interaction systems in Zhoushani and its four regions are in a coordinated development stage, with relatively stable relations between the social, economic and ecological systems. The vulnerability coordination levels of the three binary interaction systems in Zhoushan peaked around 2018, after which their values showed a downward trend. After 2020, the three binary interaction systems gradually enter a level of disorder with significant fluctuations. The synergistic effect of developing the economic-social, economic-ecological, and social-ecological systems decreased, which to some extent constrained the coordinated development of the economic-social-ecological ternary system in Zhoushan.

From the ternary structure analysis, it is concluded that the vulnerability coordination degree of the economic-social-ecological ternary system in Zhoushan from 2011 to 2022 underwent a transition from high-quality coordination to good coordination to intermediate coordination to moderate disorder to severe disorder, with synergistic effect gradually deteriorating. Specifically, in Dinghai, the vulnerability coordination degree of the economic-social-ecological ternary system evolves from good coordination to intermediate coordination to bare coordination to mild disorder; in Putuo, it evolves from good coordination to intermediate coordination to mild disorder; in Daishan, it evolves from intermediate coordination to primary coordination to bare coordination; in Shengsi, it evolves from primary coordination to bare coordination to mild disorder. Dinghai reached its highest degree of coordinated development (0.878) in 2017, while the highest values in Putuo, Daishan, and Shengsi occurred in 2017 (0.801), 2011 (0.739), and 2013 (0.684), respectively. From the highest values to 2022, Putuo experienced the most significant decline in its degree of coordinated development, dropping from 0.801 to 0.304, a decrease of approximately 62.1%, followed by Dinghai, which declined from 0.878 to 0.369, a decline of about 57.9%. Similar to the binary systems, the ternary systems in each district and county were at a level of coordination before 2020, after which their indices evolved to a level of disorder.

### 3.4. Analysis of influencing factors of vulnerability

#### 3.4.1. Obstacle degree at the system level.

**Fig 7:**
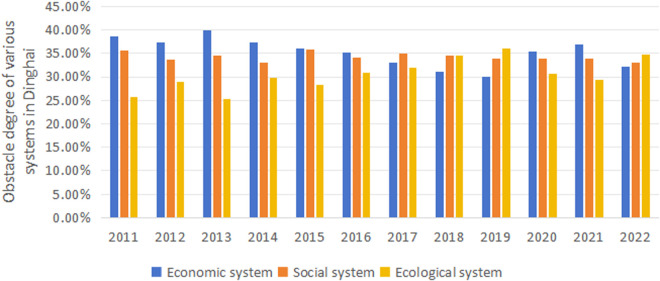
Changes in the degree of systemic obstacle in Dinghai.

As shown in Fig 7, the obstacle degree of the economic system in Dinghai varies from 38.67% in 2011 to 32.22% in 2022, exhibiting a downward tendency with some fluctuations, especially peaking at 36.86% in 2021; the social system’s obstacle degree dropped from 35.69% in 2011 to 33.10% in 2022, with minor overall changes, signifying a relatively stable influence on regional development; the ecological system’s obstacle degree escalates notably from 0.256 in 2011 to 34.68% in 2022, reaching its apex and of 36.08% in 2019, becoming the most substantial hindrance among the three systems to the regional development. Despite the fluctuations in the economic and social system obstacles, their overall shifts are modest, with the social system demonstrating more excellent stability. The ecological system’s obstacle degree consistently climbs, eventually surpassing the economic system as the main contributor to the systemic challenge.

**Fig 8:**
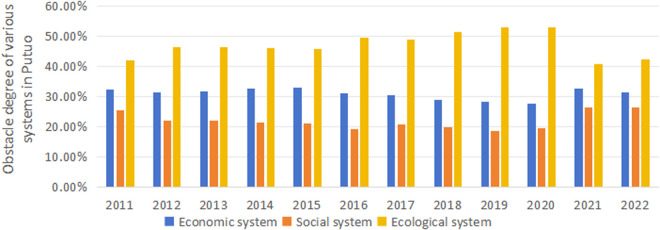
Changes in the degree of systemic obstacle in Putuo.

According to Fig 8, it can be observed that from 2011 to 2022, the order of the obstacle degree in the complex system of Putuo is mainly ecological system > economic system > social system. Specifically, the ecological system’s obstacle degree is 42.05% in 2011, showing an upward trend from 2012 to 2019, reaching its peak at 53.11% in 2019. Although it declines slightly afterward, it rises again to 42.26% in 2022. This indicates that the ecological system faces significant pressure despite short-term fluctuations. The economic system’s obstacle degree fluctuates from 32.47% in 2011 and decreases to 31.36% in 2022, showing an overall downward trend despite some fluctuations. The social system’s obstacle degree gradually decreases from 25.48% in 2011 to its lowest point of 18.67% in 2019, then increases to 26.38% in 2022, presenting a trend of first decreasing and then increasing. The obstacle degrees of Putuo’s economic, social, and ecological systems have varied over time, with a certain degree of fluctuation. Still, the ecological system’s obstacle degree has consistently remained high. From a long-term perspective, the problems existing in the ecological system of Putuo are particularly prominent in its overall development.

**Fig 9:**
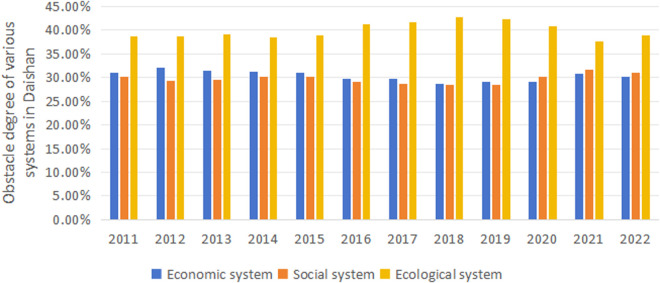
Changes in the degree of systemic obstacle in Daishan.

As shown in Fig 9, from 2011 to 2022, there are dynamic changes in the obstacle degrees among the economic, social, and ecological systems in Daishan. The ecological system has the highest degree of obstacle, while the economic and social systems fluctuate over the years without significant increases overall. Specifically, the ecological system’s obstacle degree gradually increases from 38.67% in 2011 to its peak of 42.80% in 2018, then slightly decreases but rises again to 38.82% in 2022. This indicates that the pressure on the ecological system has increased despite some relief in recent years. The economic system’s obstacle degree fluctuates slightly throughout the period, decreasing slightly from 31.07% in 2011 to 29.10% in 2020, then rising again to 30.08% in 2022, showing a relatively stable overall trend. The social system’s obstacle degree gradually increases from 0.3026 in 2011 to its highest point of 0.3170 in 2021, then slightly decreases to 0.3110 in 2022. In summary, Daishan’s economic system is relatively stable, but the obstacle degrees of the ecological and social systems show an upward trend, particularly the ecological system, which faces considerable pressure.

**Fig 10:**
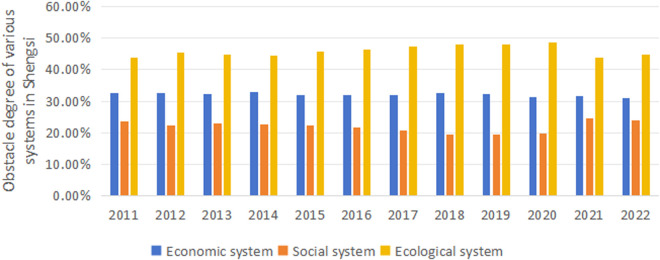
Changes in the degree of systemic obstacle in Shengsi.

In Shengsi County, from 2011 to 2022 (as shown in Fig 10), the ecological system’s obstacle degree increases yearly from 0.4396 in 2011, reaching its peak of 0.4864 in 2020. Although it decreases slightly afterward, it maintains a relatively high level of 0.4481 in 2022. This indicates that the ecological system’s impact on the overall system shows an increasing trend during this period. The economic system’s obstacle degree remains relatively stable throughout the period, slightly decreasing from 0.3254 in 2011 to 0.3106 in 2022, indicating that the economic system’s obstacle degree does not change significantly. The social system’s obstacle degree gradually decreases from 0.2351 in 2011 to its lowest point of 0.1943 in 2019, then substantially increases to 0.2452 in 2021, followed by a slight decrease to 0.2413 in 2022. In comparison, Shengsi’s ecological system has the highest overall obstacle degree and shows a clear growth trend, indicating that Shengsi faces significant pressure from the ecological system during this period. The degree of economic system obstacle is relatively low and remains stable, suggesting that economic activities have a comparatively more minor impact on the overall system. Although the degree of obstacles in the social system is relatively low and decreases yearly before 2019, it has significantly increased in recent years.

#### 3.4.2. Obstacle degree of indicator layers in each region.

Calculating the obstacle degree of economic-social-ecological complex system vulnerability indicators in various counties of Zhoushan from 2011 to 2022 and ranking the top 5 indicators with the highest obstacle degree each year shows the specific results in Table 5.

**Table 5:** Obstacle Factors in Reducing the Vulnerability in Zhoushan.

Region	Rank	2011	2012	2013	2014	2015	2016	2017	2018	2019	2020	2021	2022
**Dinghai**	1	A10	A10	A10	A10	A10	A10	A10	A10	A10	A10	A3	A3
	2	A3	A3	A3	A3	C9	C9	C9	C9	C9	C9	B1	B1
	3	B1	B1	B1	B1	B1	B1	B1	B1	B1	B1	B3	B3
	4	B3	C9	C9	C9	B3	B3	B3	B3	B3	B3	C9	C9
	5	C9	B3	B3	B3	A3	C3	C11	C11	C11	A3	A1	A1
**Putuo**	1	C7	C7	C7	C7	C7	C7	C7	C7	C7	C7	C7	C7
	2	C4	C4	C4	C4	C4	C4	C4	C4	C4	C4	A3	A3
	3	A3	A3	A3	A10	A10	A10	A10	A10	A10	A10	C4	C4
	4	A10	A10	A10	A3	A3	A3	A3	A3	C3	A3	A10	A10
	5	B1	C9	C9	C9	C9	C3	C3	C3	A3	C3	B1	B1
**Daishan**	1	C7	C7	C7	C7	C7	C7	C7	C7	C7	C7	C7	C7
	2	A3	A3	A3	A10	A10	A10	A10	A10	A10	A10	A3	A3
	3	C4	C4	A10	A3	C4	C4	C4	C4	C4	C4	A10	A10
	4	A10	A10	C4	C4	A3	A3	A3	A3	A3	A3	C4	C4
	5	B1	B1	B1	B1	B1	B1	B1	B1	B1	B1	B1	B1
**Shengsi**	1	C7	C7	C7	C7	C7	C7	C7	C7	C7	C7	C7	C7
	2	C4	C4	C4	C4	C4	C4	C4	C4	C4	C4	C4	C4
	3	A3	A3	A3	A3	A10	A3	A10	A10	A10	A10	A3	A3
	4	A10	A10	A10	A10	A3	A10	A3	A3	A3	A3	A10	A10
	5	B3	C2	C2	C1	C2	C1	C1	C1	C1	C1	B1	B1

In the economic-social-ecological complex system of Dinghai, factor A10 has been one of the indicators with the highest degree of obstacle from 2011 to 2020. Indicator A3 has frequently appeared in the list of obstacle factors since 2011, especially becoming the primary obstacle factor in 2021 and 2022. Additionally, B1, B3, and C9 have appeared in the top five obstacle factors of Dinghai for many years. In the early period (2011–2020), the Total import and export trade of islands has always been the main obstacle factor. Starting from 2021, the Density of the tourism economy has risen to become the primary obstacle factor, which may indicate that the development of the tourism industry has become a critical bottleneck for local economic growth, and the disturbance and pressure brought by tourism activities have become the main vulnerability factors of the complex system in Dinghai.

From 2011 to 2022, the factors frequently appearing in the top five obstacle factors of the complex system in Putuo are C7, C4, A3, A10, and C3. From 2011 to 2014, the Island land and sea area coefficient and the Proportion of construction land were consistently the main obstacle factors, while the Density of the tourism economy and Total import and export trade of islands also frequently appear, indicating that natural conditions, land use, tourism, and foreign trade are the main obstacle factors faced by Putuo. From 2015 to 2020, in addition to the above factors, Electricity consumption per unit GDP begins to appear frequently, indicating that the impact of energy efficiency on Putuo gradually increased. From 2021 to 2022, the Density of the tourism economy rises to become the main obstacle factor, which may suggest that the development of the tourism industry requires more attention.

Among the top five obstacle factors in the complex system of Daishan, factor C7 has been the main obstacle factor from 2011 to 2022, similar to Putuo, indicating that the geographical and natural conditions of the region have posed certain limitations on its development. Secondly, factor A3 has been frequently identified as an obstacle factor over the years; in addition, C4, A10, and B1 are also major obstacle factors in the development of Daishan.

In the economic-social-ecological complex system of Shengsi, factor C7 has been the main obstacle from 2011 to 2022, indicating that geographical and natural conditions have limited the development of Shengsi. Next is C4, which has been the second-ranked obstacle factor in the complex system of Shengsi for 12 consecutive years. The frequent appearance of this factor indicates that land use efficiency and rational planning of construction land may be among the crucial obstacles Shengsi faces. Then, A3 and A10 in the economic system fluctuate between the third and fourth obstacle degree factors during 2011–2022. Finally, C2 and C1 in the ecological system alternately appear from 2012 to 2020, indicating that the ecological problems in Shengsi are more prominent during this period. However, after 2020, B1 in the social system replaced C1 and C2 as the fifth obstacle factor.

Overall, A10, A3, C4, C7, and B1 are the main obstacle factors for the vulnerability of the economic-social-ecological complex system in the Zhoushan during 2011–2022, appearing 46 times, 44 times, 36 times, 36 times, and 29 times respectively, indicating that the above indicators are the main factors affecting the vulnerability level of the system in Zhoushan. It can be seen that enhancing the openness of islands, rationally planning the development and utilization of tourism resources, alleviating land use pressure, improving the utilization efficiency of island resources, and reasonably controlling tourist carrying capacity are the main tasks to reduce the vulnerability of the island tourism destination system.

## 4. Conclusions and discussion

### 4.1. Conclusions

Based on the above data analysis, the main conclusions are as follows:

Firstly, the vulnerability changes of the economic, social, and ecological subsystems in each region in Zhoushan exhibit different characteristics. The vulnerability of the economic and social subsystems shows a significant downward trend, indicating that their ability to resist external disturbances continuously increases. In comparison, the vulnerability index of the ecological subsystem changes relatively smoothly. From the perspective of the complex system vulnerability, although the vulnerability index of each region generally shows a downward trend, there are specific differences in the paths and degrees of change: Dinghai, despite having the highest vulnerability index, shows a continuous downward trend; Putuo experiences an inverted U-shaped evolution process of rising first and then falling; Daishan’s vulnerability index rebounds slightly in recent years while showing an overall decline; Shengsi continues to decrease and turns from positive to negative, exhibiting a favorable development trend. In addition, from the perspective of spatial evolution, low-vulnerability areas are gradually replacing medium-high-vulnerability areas. Specifically, Dinghai has transformed from a high-vulnerability state to a medium-vulnerability state. Putuo has also completed the evolution from medium vulnerability to low vulnerability, while Daishan and Shengsi have always maintained a relatively stable low-vulnerability state.

Secondly, the results of the system-coordinated analysis show that the coordination degree of the binary interaction systems of economic-social-ecological in Zhoushan has undergone a process of transformation from well-coordinated to uncoordinated, with the most significant change in the coordination degree of the economic-social system. From a regional perspective, the coordination status of the binary interaction systems in various districts and counties has also mostly evolved from a coordinated level to an uncoordinated level. Regarding the degree of vulnerability coordination in the ternary system, Zhoushan has experienced a transformation from high-quality coordination to extreme disorder, and the synergistic effect among the subsystems has gradually weakened. The coordination degree of the ternary system in various districts and counties has also shown varying degrees of decline. Based on the system coordination degree analysis, when formulating relevant policies, Zhoushan needs to focus on controlling the threshold of a single system and pay more attention to the coordinated development of the complex systems of economy, society, and ecology.

Thirdly, the obstacle model analysis shows that the obstacle degree of the ecological system in Dinghai presents an upward trend, gradually replacing the economic system as the most critical factor affecting the vulnerability of the complex system in the area from 2011 to 2022; the ecological system obstacle degree in Putuo has always been at a high level; the ecological and social system obstacle degrees in Daishan continue to rise; the ecological system obstacle degree in Shengsi ranks first among the four regions and shows a clear growth trend. At the indicator level, the Total import and export trade of the islands, the Density of the tourism economy, the Proportion of construction land, the land-sea area coefficient of the islands, and Tourist density are the key obstacle factors affecting the system vulnerability of Zhoushan. To effectively reduce the system vulnerability of island tourism destinations, Zhoushan urgently needs to take targeted policy measures from the aspects of improving the level of opening to the outside world, optimizing the development and layout of tourism resources, alleviating the pressure of land resources, improving the utilization efficiency of island resources, and reasonably controlling the carrying capacity of tourists.

### 4.2. Discussion

The analysis of the vulnerability of the economic-social-ecological complex system of island tourist destinations provides a new perspective for studying the man-land relationship of island tourist destinations. By integrating the interaction of economic, social and ecological systems, this study uses quantitative analysis methods to comprehensively assess the vulnerability of island tourism destinations. At the same time, this study also discusses the key factors affecting the vulnerability of island tourism destinations, and puts forward strategic suggestions accordingly, aiming to provide theoretical and practical basis for reducing the vulnerability of island areas.

Previous studies mainly explored the vulnerability of island areas from the aspects of ecological environment and economic single system [46,47], but did not analyze the vulnerability of multi-dimensional system in the context of island tourism development. This study takes Zhoushan Islands as an example to analyze the vulnerability of Zhoushan ‘s economic-social-ecological complex system under the background of island tourism development. It is not only a powerful exploration of the resilience of the complex system induced by tourism activities, but also a supplement to the study of island sustainable development. On the macro level, like previous studies, this study points out that the coupling and coordinated development of economy, society and ecology is an important path to achieve sustainable development, and the imbalance of any one subsystem will bring about the negative effect of the overall coordinated development [48]; as the resource base of island tourism development, the imbalance of ecological system will significantly bring about the coordinated development of social and economic systems. Therefore, the island development state with ecological environment protection as the core is an important aspect of maintaining the resilience of island economic and social development. In addition, the study found that for island development, basic living resources (such as energy) and objective limitations of development (such as land) are still obstacles to the stability of island systems. However, the study also shows that land-sea coordination provides opportunities for island development, strengthens the spatial relationality with the ocean as the link, and vigorously develops regional marine economy and global marine trade, which helps to weaken the system vulnerability risk induced by resource constraints.

However, there are still some limitations in this study. Due to the multidimensionality and complexity of vulnerability, it is still a challenge to fully integrate economic, social and ecological factors into the evaluation system. This study focuses on the use of social development data to measure the vulnerability of complex systems, but micro-social interaction is equally important. Future research should adopt more qualitative and quantitative research methods to explore the interaction mode between different subjects such as community residents, tourists and tourism enterprises under the background of tourism development, so as to obtain a more comprehensive analysis of the vulnerability of island tourism destinations.

## Supporting information

S1 File Data. (XLSX)
